# Supplementary material for: Nucleotide variation and balancing selection at the Ckma gene in Atlantic cod: analysis with multiple merger coalescent models
Source: PeerJ. 2015 Feb 24;3:e786. doi: 10.7717/peerj.786 (PMC4349156; doi:10.7717/peerj.786)
Supplement: Table S1 [file peerj-03-786-s014.pdf]

Table S1. Primer sequences for amplification and sequencing fragments of *Ckma* gene from Atlantic cod and closely related taxa.

Primer name	Use	Sequence
creL8945	Amplification	5′-GTT TAG GAA TCT ACG CCC ATC CAG AGA CA-3′
creR12945	Amplification	5′-TGG CTA TCA TGC ATT CCC AAT GTT C-3′
creseqR12388	Sequencing	5′-CAT GAC CGT TGG CTG CGT TG-3′
creseqL10486	Sequencing	5′-TCG AAC ACT CCA CCG ACG GA-3′
creseqR10602	Sequencing	5′-ACA GAT TTC GTC TGC CGA GT-3′
