# Supplementary material for: Nucleotide variation and balancing selection at the Ckma gene in Atlantic cod: analysis with multiple merger coalescent models
Source: PeerJ. 2015 Feb 24;3:e786. doi: 10.7717/peerj.786 (PMC4349156; doi:10.7717/peerj.786)
Supplement: Table S3 — Divergence, D, and standard deviation, s, found using Jukes and Cantor correction. [file peerj-03-786-s016.pdf]

Table S3. Gross D_{xy} and net D_a nucleotide divergence per site between *Gadus morhua* Gmo and *Gadus macrocephalus* Gma and *Gadus chalcogrammus* Gch and between *A* and *B* alleles of Atlantic cod.

Gene	Comparison	D_{xy}	$s_{D_{xy}}$	D_a	s_{D_a}
HbA2	Gmo vs Gma	0.013	0.004	0.012	0.004
HbA2	Gmo vs Tch	0.017	0.014	0.017	0.014
Myg	Gmo vs Gma	0.027	0.007	0.025	0.007
Ckma	Gmo vs Gma	0.014	0.001	0.011	0.001
Ckma	Gmo vs Tch	0.015	0.003	0.013	0.003
Ckma	*A* vs *B*	0.008	0.0005	0.006	0.0005

Divergence, D, and standard deviation, s, found using Jukes and Cantor correction.
