# Supplementary material for: Nucleotide variation and balancing selection at the Ckma gene in Atlantic cod: analysis with multiple merger coalescent models
Source: PeerJ. 2015 Feb 24;3:e786. doi: 10.7717/peerj.786 (PMC4349156; doi:10.7717/peerj.786)
Supplement: Table S4 — Individuals with species and locality codes, first aa represents majority and the second the change, position refers to position in concatenated sequence in Table S2. [file peerj-03-786-s017.pdf]

Table S4. Non-synonymous changes within and between species.

Individual	amino acids	position
100896.Gmo.Gre	M ⇔ T	37
117937.Gmo.Ice	I ⇔ T	469
152915.Gmo.Nor	S ⇔ G	1158
118708.Gmo.Ice	R ⇔ G	1182
152978.Gmo.Nor	E ⇔ G	2068
152066.Gma.Pac	I ⇔ T	469
152047.Gma.Pac	V ⇔ I	2094
104474.Bsa.Gre	R ⇔ G	1182
104725.Bsa.Gre	Q ⇔ G	1305, 1306, 1307
104474.Bsa.Gre	Q ⇔ G	1305, 1306, 1307

Individuals with species and locality codes, first aa represents majority and the second the change, position refers to position in concatenated sequence in Table S2.
