# Supplementary material for: Nucleotide variation and balancing selection at the Ckma gene in Atlantic cod: analysis with multiple merger coalescent models
Source: PeerJ. 2015 Feb 24;3:e786. doi: 10.7717/peerj.786 (PMC4349156; doi:10.7717/peerj.786)
Supplement: Table S5 — Test is twice the ln L difference of the two models, neutrality and selection at Ckma. Three loci are under test: Hemoglobin α2 (Hbα2), Myoglobin (Myg), and Creatine Kinase Muscle (Ckma). θ is the scaled effective population size and the parameter k measures changes in diversity due to selection. Based on method of Wright and Charlesworth (2004). [file peerj-03-786-s018.pdf]

Table S5. Maximum likelihood analysis of a Kingman-coalescent HKA test of neutrality and selection at three genes in Atlantic cod.

					Hbα2		*Myg*		*Ckma*	
Description	$\ln L$	T	Test	*df*	θ	k	θ	k	θ	k
Neutral, all $k = 1$	-18.66	2.46			0.0035	1	0.0068	1	0.0056	1
Selection at *Ckma*	-17.47	3.86	2.38	1	0.0029	1	0.0054	1	0.0032	2.12

Test is twice the $\ln L$ difference of the two models, neutrality and selection at *Ckma*. Three loci are under test: Hemoglobin α 2 (*Hbα2*), Myoglobin (*Myg*), and Creatine Kinase Muscle (*Ckma*). θ is the scaled effective population size and the parameter k measures changes in diversity due to selection. Based on method of Wright and Charlesworth (2004).
