# Supplementary material for: Nucleotide variation and balancing selection at the Ckma gene in Atlantic cod: analysis with multiple merger coalescent models
Source: PeerJ. 2015 Feb 24;3:e786. doi: 10.7717/peerj.786 (PMC4349156; doi:10.7717/peerj.786)
Supplement: Table S6 [file peerj-03-786-s019.pdf]

Table S6. Frequency of *A* and *B* alleles in different localities.

	Can	Gre	Ice	Nor	Bar	Whi	Far	Nse	Bal	Cel	Iri	**Sum**
A allele	0	0	7	0	0	1	7	8	7	7	6	**43**
B allele	9	5	45	13	5	1	0	1	0	0	0	**79**
Sum	9	5	52	13	5	2	7	9	7	7	6	**122**
