# Supplementary material for: Nucleotide variation and balancing selection at the Ckma gene in Atlantic cod: analysis with multiple merger coalescent models
Source: PeerJ. 2015 Feb 24;3:e786. doi: 10.7717/peerj.786 (PMC4349156; doi:10.7717/peerj.786)
Supplement: Table S7 — Probabilities in black on upper triangular, boldface are significant P values. North (blue) and South (red) defined ad hoc by results. [file peerj-03-786-s020.pdf]

Table S7. Pairwise F_{ST} values (lower triangular) of population differentiation among localities.

	Can	Gre	Ice	Nor	Bar	Far	Nse	Bal	Cel	Iri
Can		0.78	0.06	0.11	0.14	**0.00**	**0.00**	**0.00**	**0.00**	**0.00**
Gre	0.04		0.22	0.55	0.12	**0.00**	**0.00**	**0.00**	**0.00**	**0.00**
Ice	0.08	0.01		0.21	0.53	**0.00**	**0.00**	**0.00**	**0.00**	**0.00**
Nor	0.03	0.00	0.02		0.13	**0.00**	**0.00**	**0.00**	**0.00**	**0.00**
Bar	0.01	−0.08	−0.02	−0.06		**0.00**	**0.00**	**0.00**	**0.00**	**0.00**
Far	0.88	0.81	0.77	0.84	0.84		0.41	0.88	0.21	0.22
Nse	0.78	0.71	0.65	0.74	0.74	0.04		0.93	0.48	0.16
Bal	0.82	0.76	0.71	0.78	0.78	−0.01	−0.03		0.36	0.82
Cel	0.91	0.84	0.80	0.87	0.87	−0.05	0.07	−0.01		0.09
Iri	0.88	0.82	0.78	0.84	0.84	0.02	0.04	0.01	0.04	

Probabilities in black on upper triangular, boldface are significant P values. North (blue) and South (red) defined ad hoc by results.
