# Supplementary material for: Nucleotide variation and balancing selection at the Ckma gene in Atlantic cod: analysis with multiple merger coalescent models
Source: PeerJ. 2015 Feb 24;3:e786. doi: 10.7717/peerj.786 (PMC4349156; doi:10.7717/peerj.786)
Supplement: Table S8 [file peerj-03-786-s021.pdf]

**Table S8.** Likelihood ratio test statistics $G$ for observed site frequency spectra of *Ckma* and expectation according to different coalescent models.

| Model | $G$ | Comparison | $2\Delta G$ | $df$ |
|---|---|---|---|---|
| I. Kingman | 149.26 | | | |
| II. Beta$(2-\alpha,\alpha)$ | 116.21 | I vs II | 66.10 | 1 |
| III. point-mass | 114.42 | I vs III | 69.69 | 1 |
