# Supplementary material for: Nucleotide variation and balancing selection at the Ckma gene in Atlantic cod: analysis with multiple merger coalescent models
Source: PeerJ. 2015 Feb 24;3:e786. doi: 10.7717/peerj.786 (PMC4349156; doi:10.7717/peerj.786)
Supplement: Table S9 [file peerj-03-786-s022.pdf]

Table S9. The ℓ_2 distance (Birkner et al., 2013b) between the observed site frequency spectra and expectation according to the Beta$(2-\alpha,\alpha)$ and point-mass multiple merger coalescent models for the three genes, *Ckma*, *HbA2*, and *Myg*.

Locus	Beta$(2-\alpha,\alpha)$	Point-mass
Ckma	7.46	8.16
HbA2	2.05	1.40
Myg	2.83	3.92
