# Supplementary material for: Nucleotide variation and balancing selection at the Ckma gene in Atlantic cod: analysis with multiple merger coalescent models
Source: PeerJ. 2015 Feb 24;3:e786. doi: 10.7717/peerj.786 (PMC4349156; doi:10.7717/peerj.786)
Supplement: Table S10 — The ℓ2 distance and approximate log Likelihood (Eldon et al., 2015) between the observed site frequency spectra and expectation according to the algebraic (A, γ) and exponential (E, β) growth models (Eldon et al., 2015) for the three genes, Ckma, HbA2, and Myg. [file peerj-03-786-s023.pdf]

Table S10. The ℓ_2 distance and approximate log Likelihood (Eldon et al., 2015) between the observed site frequency spectra and expectation according to the algebraic $(\mathbf{A}, \gamma)$ and exponential $(\mathbf{E}, \beta)$ growth models (Eldon et al., 2015) for the three genes, *Ckma*, *HbA2*, and *Myg*.

	$\mathbf{A}, \gamma$		$\mathbf{E}, \beta$	
Locus	ℓ_2	$\log L$	ℓ_2	$\log L$
Ckma	10.09	154.11	9.32	165.83
HbA2	6.95	11.21	6.87	10.16
Myg	2.43	51.98	2.83	50.44
