# Supplementary material for: Nucleotide variation and balancing selection at the Ckma gene in Atlantic cod: analysis with multiple merger coalescent models
Source: PeerJ. 2015 Feb 24;3:e786. doi: 10.7717/peerj.786 (PMC4349156; doi:10.7717/peerj.786)
Supplement: Table S2 — The top panel of the boxhead indicates 5′ UTR, exon, intron, and 3′ UTR location of segregating sites, numbers represent exon and intron numbers, s, n, -, u, and ∇ represent synonymous, nonsynonymous, intronic, UTR, and indel sites respectively. The middle panel of the boxhead are site numbers of the Ckma gene (3,604 bp) and the bottom panel are site numbers of the concatenated 2,500 bp sequenced fragment (Fig. S2). Horizontal lines are drawn between A and B alleles and between Atlantic cod and closely related species Gma Gadus macrocephalus, Gch Gadus chalcogrammus, Gog Gadus ogac, and Bsa Boreogadus saida. Locality codes are as indicated in methods. ∇1 is a four bp indel at sites 573–576. ∇2 is an 11 bp indel at sites 941–951. ∇3 is a two bp indel at sites 1,470–1,471. ∇4 is an 14 bp indel at sites 1,828–1,841. ∇5 is an three bp indel at sites 2,127–2,129. ∇6 is a one to eight bp indel at sites 2,217–2,224. ∇7 is a one bp indel at site 2,354. ∇8 is a one bp indel at site 2,476. The Moen et al.Gm366-0514 locus with an FST = 0.83 is at site 1,634, the Gm366-1022 locus with an FST = 0.82 is at site 2,426, and the Gm366-1073 with an FST = 0.82 is at site 2,476. A alleles are in red and B alleles in blue. Closely related species are colored: Gma is green, Gog is darkgreen, Gch is darkred, and Bsa is darkblue. [file peerj-03-786-s015.pdf]

Table S2 Segregating sites of the *Ckma* gene among 122 Atlantic cod individuals and 10 individuals of closely related species.[a]

	55eeeeeeeiiiiiiiiiiiiiiiieeeeeiiii	i	iiiiiiiiiiiiiiiiiiiiiiieeeeeiiiiiiii	i	iiiiiiiiiiiiiieeeeeeeeiiiiii	i	ieeeeeiiiiiiiiiiiii	i	iiiiiiiieeeeee33	3	33333	3	3333333	3	333333	3	3
	''11111111111111111111111222222222	2	222222222222222222222223333333333333	3	3333333333333344455555555555	5	5666666666666666666	6	66666666777777''	'	'''''	'	'''''''	'	''''''	'	'
	uunssssss----------------ssnss----	-	-----------------------sssss--------	-	--------------nnsnnnss------	-	-sssss-------------	-	--------sssnnsuu	u	uuuuu	u	uuuuuuu	u	uuuuuu	u	u
		▽		▽		▽		▽		▽		▽		▽		▽	
		1		2		3		4		5		6		7		8	
	11111111	1	111111111111111111111111111111111111	1	1111111111111111122222222222	2	2222222222222222222	2	2222222222222333	3	33333	3	3333333	3	333333	3	3
	5566777778888888888889999900001111	1	111111111222222222222222222344444444	5	5555555666666677700001111222	2	2223344445555555555	6	6666677789999000	0	00000	1	1111222	2	222333	3	3
	7808455691122233368990124813671223	4	444566689001222223445556677511345669	1	2356678112446924588881558001	4	4581206880113455669	1	2257822362458011	1	33667	0	1799224	4	555012	6	8
	6027703521223505759678792114849072	1	345625969085125788580683858035294191	1	6282307055035037501285458349	6	7299397497476801277	6	7949102408373056	7	69455	9	7229571	3	147558	5	3
					11111111111111111111111	1	1111111111111111111	1	1111111112222222	2	22222	2	2222222	2	222222	2	2
	1111222222222233333333444555555	5	555556666666666666666666677778888899	9	9999900000001111133333334444	4	4455566777777777778	8	8889999990000111	1	11111	2	2233333	3	333444	4	4
	1132888024455667702334567146005566	7	778990023345556667888999011845688902	4	6699902456770258900014781224	7	7714439013346777892	4	5570044573569122	2	45778	2	2801335	5	666123	7	9
	1572258076778050204123247669394527	6	890170414530670233035138303580749646	6	1737852500580582056730903894	1	2744842942921356722	1	2494657919484167	8	70566	0	8330682	4	258669	6	4
119233.Gmo.Ice	cttcctttagtcaatcgattttacccctcacaag	a	caacctatctaattaattataaccattcttttaaaa	t	ttgacattctgtataatcagccctgcgc	t	ctccctgcgcgctaagttg	a	agcaaacgttgagcaa	a	ttatt	g	ttcggta	-	gatttt	t	c
140397.Gmo.Bal	....	.	....	.	....	.	....	.	....	.	.....	.	....	.	......	.	.
140380.Gmo.Bal	....	.	....	.	....	.	....	.	....	.	.....	.	....	.	......	.	.
140163.Gmo.Cel	....	.	....	.	....	.	....	.	....	.	.....	.	....	.	......	.	.
105686.Gmo.Nse	....	.	....	.	....	.	....	.	....	.	.....	.	....	.	......	.	.
106287.Gmo.Nse	....	.	....	.	....	.	....	.	....	.	.....	.	....	.	......	.	.
114718.Gmo.Far	....	.	....	.	....	.	....	.	....	.	.....	.	....	.	......	.	.
106632.Gmo.Nse	....	.	....	.	....	.	....	.	....	.	.....	.	....	.	......	.	.
140174.Gmo.Cel	....	.	....	.	....	.	....	.	....	.	.....	.	....	.	......	.	.
115404.Gmo.Far	....	.	....	.	....	.	....	.	....	.	.....	.	....	.	......	.	.
300006.Gmo.Iri	....	.	....	.	....	.	....	.	....	.	.....	.	....	.	......	.	.
300007.Gmo.Iri	.........a........................	g	....	.	....	.	.................c.	.	....	.	.....	.	....	.	......	.	.
105661.Gmo.Nse	.........................t........	.	........t...........................	.	....	.	....	.	....	.	.....	.	....	.	......	.	.
300002.Gmo.Iri	....	.	......................t.............	.	....	.	....	.	....	.	.....	.	....	.	......	.	.
140384.Gmo.Bal	....	.	....	.	....	.	.......t...........	.	....	.	.....	.	....	.	......	.	.

continued on next page

```
               55eeeeeeeiiiiiiiiiiiiiiiieeeeeiiii i iiiiiiiiiiiiiiiiiiiiiiieeeeeiiiiiiii i iiiiiiiiiiiiiieeeeeeeeiiiiii i eeeeeiiiiiiiiiiiiii i iiiiiiiieeeeee33333333 3 3333333 3 333333 3 3
               ''11111111111111111111111222222222 2 222222222222222222222222333333333333 3 3333333333333333444555555555 5 5666666666666666666 6 66666666777777'''''''' ' ''''''' ' '''''' ' '
               uussssss-----------------sssss---- - -----------------------sssss-------- - -------------sssssss-------- - -sssss------------- - --------ssssssuuuuuuuu u uuuuuuu u uuuuuu u u
                                                  ▽                                      ▽                              ▽                     ▽                ▽       ▽         ▽        ▽
                                                  1                                      2                              3                     4                5       6         7        8

                                         11111111 1 111111111111111111111111111111111111 1 1111111111111111122222222222 2 2222222222222222222 2 2222222222222233333333 3 3333333 3 333333 3 3
               5566777778888888888889999900001111 1 111111111222222222222222222344444444 5 5555555666666667770000111122 2 2223344455555555555 6 6666677789999000000000 1 1111222 2 222333 3 3
               7808455691122233368990124813671223 4 444566689001222223445556677511345669 1 2356678112446924588881558001 4 4581206880113455669 1 2257822362458011133667 0 1799224 4 555012 6 8
               6027703521223505759678792114849072 1 345625969085125788580683858035294191 1 6282307055035037501285458349 6 7299397497476801277 6 7949102408373056769455 9 7229571 3 147558 5 3

                                                                                              11111111111111111111111 1 1111111111111111111 1 1111111122222222222222 2 2222222 2 222222 2 2
                  1111222222222223333333334445555 5 555556666666666666666666677778888899 9 9999900000011113333333334444 4 4455566777777777778 8 8889999990000111111111 2 2233333 3 333444 4 4
               1132888024455667702334567146005566 7 778990023345556667888999011845688902 4 6699902456770258900014781224 7 7714439013346777892 4 5570044573569122245778 2 2801335 5 666123 7 9
               1572258076778050204123247669394527 6 890170414530670233035138303580749646 6 1737852500580582056730903894 1 2744842942921356722 1 2494657919484167870566 0 8330682 4 258669 6 4
140170.Gmo.Celt .................................. . .................................... . ............................ . ................... . ...................... . ....... . ...... . .
114780.Gmo.Far  .................................. . .................................... . ............................ . ................... . ...................... . ....... . ...... . .
140193.Gmo.Cel  .................................. . .................................... . ...........c................ . ................... . ...................... . ....... . ...... . .
300005.Gmo.Iri  ..........................g....... . .................................... . .c.......................... . ................... . ...................... . ....... . ...... . .
300004.Gmo.Iri  .................................. . .................................... . ............................ . ................... . ...................... . ....... . ..c... . .
140383.Gmo.Bal  .................................. . .....................t.............. . ..a......................... . ................... . ...................... . ....... . ...... . .
114844.Gmo.Far  .................................. . ....a............................... . ............................ . ................... . ...................... . ....... . ...... . .
140381.Gmo.Bal  .................................. . .................................... . ............................ - ................... g ...................... . ....... . ...... . .
115674.Gmo.Far  .................................. . ..............t..................... . ............................ . ..............ga... . ...................... . ....... . ...... . .
140185.Gmo.Cel  .................................. . .................................... . ............................ . ................... . ...................... . ....... . ...... . .
106284.Gmo.Nse  .................................. . .................................... . ............................ . ................... . .....g................ . ....... . ...... . .
105652.Gmo.Nse  ............................t..... . .................................... . ............................ . ................... . ...................... . ....... . ...... . .
300003.Gmo.Iri  .................................. . .................................g.. . ............................ . ................... . ...................... . ....... . ...... . .
118770.Gmo.Ice  .................................. . .................................... . ........a................t.. . ................... . ...................... . ....... . ...... . .
140419.Gmo.Whi  .................................. . .................................... . ........a................t.. . ................... . ...................... . ....... . t..cc. . .
140176.Gmo.Cel  .................................. . .................................... . ........a................t.. . ................... . ...................... . ....... . ...... c .
115596.Gmo.Far  .................................. . ......g............................. c ........a................t.. . ............c...... . ...................... . ....... . ...... . .
```

continued on next page

	55eeeeeeeiiiiiiiiiiiiiiiieeeeeiiii	i	iiiiiiiiiiiiiiiiiiiiiiieeeeeiiiiiiii	i	iiiiiiiiiiiiieeeeeeeeiiiiiii	i	ieeeeeiiiiiiiiiiiii	i	iiiiiiiieeeeee33333333	3	3333333	3	333333	3	3
	''11111111111111111111111222222222	2	22222222222222222222222333333333333 3	3	3333333333333344455555555555	5	5666666666666666666	6	66666666777777''''''''	'	'''''''	'	''''''	'	'
	uusssssss----------------sssss----	-	-----------------------sssss--------	-	--------------sssssss-------	-	-sssss-------------	-	--------ssssssuuuuuuuu	u	uuuuuuu	u	uuuuuu	u	u
		▽		▽		▽		▽	▽	▽		▽		▽	
		1		2		3		4	5	6		7		8	
	11111111	1	111111111111111111111111111111111111	1	1111111111111111122222222222	2	2222222222222222222	2	2222222222222333333333	3	3333333	3	333333	3	3
	5566777778888888888888899999000011111	1	111111111222222222222222222344444444	5	5555555666666677700001111222	2	2223344445555555555	6	6666677789999000000000	1	1111222	2	222333	3	3
	7808455691122233368990124813671223	4	444566689001222223445556677511345669	1	2356678112446924588881558001	4	4581206880113455669	1	2257822362458011133667	0	1799224	4	555012	6	8
	6027703521223505759678792114849072	1	345625969085125788580683858035294191	1	6282307055035037501285458349	6	7299397497476801277	6	7949102408373056769455	9	7229571	3	147558	5	3
					11111111111111111111111	1	1111111111111111111	1	1111111112222222222222	2	2222222	2	222222	2	2
	1111222222222233333333444555555	5	555556666666666666666666777788888899	9	9999900000001111133333334444	4	4455566777777777778	8	8889999990000111111111	2	2233333	3	333444	4	4
	1132888024455667702334567146005566	7	778990023345556667888999011845688902	4	6699902456770258900014781224	7	7714439013346777892	4	5570044573569122245778	2	2801335	5	666123	7	9
	1572258076778050204123247669394527	6	890170414530670233035138303580749646	6	1737852500580582056730903894	1	2744842942921356722	1	2494657919484167870566	0	8330682	4	258669	6	4
140179.Gmo.Cel	....	.	....	.	........a................t..	.	....	.	....	.	....	.	......	.	.
140394.Gmo.Bal	....	.	....	.	........a................t..	.	....	.	....	.	....	.	......	.	.
114528.Gmo.Far	....	.	....	.	.........................t..	.	....	.	....	.	....	.	......	.	.
105698.Gmo.Nse	....	.	t.............g..........c..........	.	....	.	....	.	....	.	....	.	......	.	.
124401.Gmo.Ice	.c...........................g....	.	t...................................	.	...........................a	.	....	.	....c.................	.	....	.	......	-	.
105657.Gmo.Nse	.............................g....	.	t...........a.g.....................	.	......a....................a	a	a..t...............	.	....	.	....	.	......	.	.
200500.Gmo.Bal	.............................g....	.	t....................t..............	.	......a...c................a	a	a....c.............	.	....	.	....	.	t...c.	-	.
118129.Gmo.Ice	.............................g....	.	t...................................	.	....	.	....	.	...................ga.	.	....	.	t...c.	-	.
119535.Gmo.Ice	.............................g....	.	t.............g......t....c.........	.	....	.	....	.	...................ga.	.	....	.	t...c.	-	.
118147.Gmo.Ice	....	.	....	.	..........c..........t......	.	....	.	.a..c..............ga.	.	....	.	t...c.	.	.
118214.Gmo.Ice	....	.	..............g......t........g.....	.	..........c.................	a	a..................	.	...................ga.	.	c......	.	t...c.	.	.
106620.Gmo.Nse	.c................................	.	t...................................	.	...........................a	a	a....c...t.........	.	.a..c..............ga.	.	....	.	t...c.	-	.
152934.Gmo.Nor	.c...........................g....	.	..............................g.....	.	......a...c.................	a	a....c...t.........	.	.a..c..............ga.	.	....	.	t...c.	.	.
119111.Gmo.Ice	.............................g....	.	t.............g...............g.....	.	......a...c.................	a	a....c...t.........	.	.a..c..............ga.	.	....	.	t...c.	.	.
118309.Gmo.Ice	.c...........................g....	.	t.............g...........c...g....t	.	......a...c.................	a	a....c...t.........	.	.a..c..............ga.	.	....	.	t...c.	-	.
140261.Gmo.Bar	.c.....................t.....g....	.	..............g....c......c...g.....	.	......a...c.................	a	a....c...t.........	.	.a..c..............ga.	.	....	.	t...c.	-	.
118705.Gmo.Ice	.c.....................t.....g....	.	............a.g...........c...g.....	.	......a...c.................	a	a....c...t.........	.	.a..c..............ga.	.	....	.	t...c.	-	.

continued on next page

```
                  55eeeeeeeiiiiiiiiiiiiiiiieeeeeiiii i iiiiiiiiiiiiiiiiiiiiiiiieeeeeiiiiiiii i iiiiiiiiiiiiiieeeeeeeeiiiiii i ieeeeeiiiiiiiiiiiii i iiiiiiiiieeeeee33333333 3 3333333 3 333333 3 3
                  ''1111111111111111111111222222222 2 22222222222222222222222233333333333333 3 33333333333333444555555555555 5 56666666666666666666 6 66666666777777'''''''' ' ''''''' ' '''''' ' '
                  uusssssss---------------sssss---- - -----------------------sssss-------- - --------------ssssssss------- - -sssss------------ - --------sssssuuuuuuuu u uuuuuuu u uuuuuu u u
                                                  ▽                                        ▽                               ▽                    ▽                ▽        ▽         ▽        ▽
                                                  1                                        2                               3                    4                5        6         7        8

                                         11111111 1 11111111111111111111111111111111111 1 11111111111111111222222222222 2 22222222222222222222 2 22222222222223333333333 3 3333333 3 333333 3 3
                  556677777888888888888899999000011111 1 1111111112222222222222222222223444444444 5 555555566666666777000011111222 2 22233444455555555555 6 666667778999900000000 1 1111222 2 222333 3 3
                  7808455691122233368990124813671223 4 44456668900122222344555667751134566 9 1 23566781124469245888815580014 4 458120688011345566 9 1 2257822362458011133667 0 1799224 4 555012 6 8
                  60277035212235057596787921148490 72 1 34562596908512578858068385803529419 1 1 6282307055035037501285458349 6 72993974974768012 77 6 7949102408373056769455 9 7229571 3 147558 5 3

                                                                                              1111111111111111111111 1 111111111111111111 1 1111111112222222222222 2 2222222 2 222222 2 2
                     111122222222222333333333444555555 5 55555666666666666666666677778888889 9 9 99999000000011111333333334444 4 44555667777777777778 8 8889999990000111111111 2 2233333 3 333444 4 4
                  11328880244556677023345671460055666 7 778990023345556667888999011845688902 4 66999024567702589000147812 24 7 771443901334677789 2 4 5570044573569122245778 2 2801335 5 666123 7 9
                  15722580767780502041232476693945 27 6 890170414530670233035138303580749646 6 17378525005805820567309038 94 1 274484294292135672 2 1 2494657919484167870566 0 8330682 4 258669 6 4

117878.Gmo.Ice.c....................t.....g.................g..........c...g.............a...c..................aa....c...tc...........a..c..............ga..........t...c.-.
118507.Gmo.Ice.c..........................g.....t............g..........c...g.............a...c...........t....aaa....c...t............a..c..............ga..........t...cc-.
200135.Gmo.Can.c..........................g.....t............g..........c...g.............a...c.................aaa....c...t............a..c..............ga..........t...c.-.
200035.Gmo.Can.c..........................g.....t............g..........c...g.............a...c.................aaa....c...t............a..c..............ga..........t...c.-.
200134.Gmo.Can.c..........................g.....t............g..........c...g.............a...c.................aaa....c...t............a..c..............ga....t.....t...c.-.
140254.Gmo.Bar.c..........................g.....t............g..........c...g.............a...c..........t.....aaa....c...t............a..c..............ga..........t...c.-.
126266.Gmo.Ice.c..........................g.....t............g..........c...g.............a...c..........t.....aaa....c...t............a..c..............ga..........t...c.-.
152938.Gmo.Nor.c..........................g.....t............g..........c...g.............a...c..........t.....aaa....c...t............a..c..............ga.-........tt..c.-.
152949.Gmo.Nor.c..........................g.....t............g..........c...g.............a...c..........t.....aaa....c..at............a..c..............ga..........t...c.-.
119545.Gmo.Ice.c..........................g..................g..........c...g.............a...c..........t.....aaa....c...tc...........a..c..............ga..........t...c.-.
200149.Gmo.Can.c..........................g.....t.......t....g..........c...g.............a...c.................aaa....c...t............a..c..............ga..........t...c.-.
119163.Gmo.Ice.c..t.......................g.....t............g..........c...g.............a...c.................aaa....c...t............a..c...........--..ga..........t...c.-.
152924.Gmo.Nor.c..........................g.....t............g..........c...g.............a...c.................aaa....c...t............a..c..............ga..........t...c.-.
118738.Gmo.Ice.c..........................g.....t............g..........c...g.............a...c.................aaa....c...t............a..c..............ga..........t...c.-.
104056.Gmo.Gre.c..........................g.....t..........a.g..........c...g.............a...c.................aaa....c...t............a..c..........ggg..ga..........t...c.-.
100896.Gmo.Gre.cc..c......................g.....t..........a.g..........c...g.............a...c.................aaa....c...t...g....a..a..c.....a.......cga..........t...c.-.
124124.Gmo.Ice.c..........................g.....t..........a.g..........c...g.............a...c.................aaa....c...t............a..c..............ga..........t...c.-.
```

continued on next page

```
                 55eeeeeeeiiiiiiiiiiiiiiiiieeeeeiiii i iiiiiiiiiiiiiiiiiiiiiiieeeeeiiiiiiii i iiiiiiiiiiiiiiieeeeeeeeiiiiii i ieeeeeiiiiiiiiiiiii i iiiiiiiiieeeeee33333333 3 3333333 3 333333 3 3
                 ''111111111111111111111112222222222 2 22222222222222222222222233333333333333 3 3333333333333344455555555555 5 566666666666666666 6 66666666777777'''''''' ' ''''''' ' '''''' ' '
                 uussssssss----------------sssss---- - -----------------------sssss-------- - --------------sssssssss------- - -sssss------------- - ---------sssssuuuuuuuu u uuuuuuu u uuuuuu u u
                                                    ▽                                        ▽                               ▽                     ▽                 ▽   ▽       ▽      ▽
                                                    1                                        2                               3                     4                 5   6       7      8

                                           11111111 1 111111111111111111111111111111111111 1 1111111111111111112222222222 2 222222222222222222 2 2222222222222333333333 3 3333333 3 333333 3 3
                 5566777778888888888888899999000001111 1 11111111112222222222222222223444444444 5 5555555666666677700001111222 2 222334444555555555 6 6666677789999000000000 1 1111222 2 222333 3 3
                 7808455691122233368990124813671223 4 444566689001222223445556677511345669 1 2356678112446924588881558001 4 458120688011345566 9 1 225782236245801113366 7 0 1799224 4 555012 6 8
                 6027703521223505759678792114849072 1 345625969085125788580683858035294191 1 6282307055035037501285458349 6 729939749747680127 7 6 794910240837305676945 5 9 7229571 3 147558 5 3

                                                                                         11111111111111111111111 1 111111111111111111 1 1111111112222222222222 2 2222222 2 222222 2 2
                    111122222222222233333333344455555 5 555556666666666666666666677778888899 9 9999900000001111133333334444 4 445556677777777778 8 8889999990000111111111 2 2233333 3 333444 4 4
                 1132888024455667702334567146005566 7 778990023345556667888999011845688902 4 6699902456770258900014781224 7 771443901334677789 2 4 557004457356912224577 8 2 2801335 5 666123 7 9
                 1572258076778050204123247669394527 6 890170414530670233035138303580749646 6 1737852500580582056730903894 1 274484294292135672 2 1 249465791948416787056 6 0 8330682 4 258669 6 4

152929.Gmo.Nor.c.........................g.....t..........a.g..........c...g...........a...c...............aaa....c...t..........a..c.............ga.........t...c.-.
117911.Gmo.Ice.c.........................g.....t..........a.g..........c...g...........a...c.........t.....aaa....c...t..........a..c.............ga......a..t...c.-.
117910.Gmo.Ice.c.........................g.....t..........a.g..........c...g...........a...c.........t.....aaa....c...t..........a..c.............ga.........t...c.-.
152919.Gmo.Nor.c.........................g.....t..........a.g..........c...g...........a...c.........t.....aaa....c...t..........a..c.............ga.........t...c.-.
119218.Gmo.Ice.c.........................g.....t..........a.g..........c...g...........a...c.........t.....aaa....c...t..........a..c.............ga.........t...c.-.
152989.Gmo.Nor.c.....c...........c.......g.....t..........a.g..........c...g...........a...c.........t.....aaa....c...t..........a..c...........c.ga.........t...c.-.
117870.Gmo.Ice.c.........................g.....t..........a.g.....t....c...g...........a...c...............aaa....c...t..........a..c.............ga.........t...c.-.
118775.Gmo.Ice.c.........................g.....t..........a.g.....t....c...g...........a...c...............aaa....c...t..........a..c.............ga.........t...c.-.
118714.Gmo.Ice.c.........................g.....t..........a.g.....t....c...g...........a...c...............aaa....c...t..........a..c.............ga.........t...c.-.
140272.Gmo.Bar.c.........................g.....t..t.......a.g.....t....ct..g...........a...c...............aaa....c...t..........a..c.............ga.........t...c.-.
140402.Gmo.Whi.c.........................g.....t..........a.g.....t....c...g...........a...c...............aaa....c...t..........a..c.............ga.........t...c.-.
118737.Gmo.Ice.c.........................g.....t............g.....t....c...g...........a...c...............aaa....c...t.t........a..c.............ga.........t...c...
152915.Gmo.Nor.c.........................g.....t............g.....t....c...g...........a...c...g...........aaa....c...t.t........a..c.............ga.........t...c.-.
200304.Gmo.Can.c.........................g.....t............g.....t....c...g...........a...c...............aaa....c...t..........a..c.............ga.a.......t...c.-.
117795.Gmo.Ice.c.........................g.....t............g.....t....c...g...........a...c...............aaa....c...t..........a..c.............ga.........t...c.-.
152933.Gmo.Nor.c.........................g.....t............g.....t....c...g...........a...c...............aaa....c...t..........a..c.............ga.........t...c.-.
200150.Gmo.Can.c.........................g.....t............g.....t....c...g...........a...c...............aaa....c...t..........a..c.............ga.........t...c.-.
```

continued on next page

```
               55eeeeeeiiiiiiiiiiiiiiiieeeeeiiiii i iiiiiiiiiiiiiiiiiiiiiiieeeeeiiiiiiii i iiiiiiiiiiiiiieeeeeeeeiiiiii i ieeeeeiiiiiiiiiiiii i iiiiiiiieeeeee33333333 3 3333333 3 333333 3 3
               ''11111111111111111111112222222222 2 222222222222222222222223333333333333 3 33333333333333444555555555555 5 5666666666666666666 6 66666666777777'''''''' ' ''''''' ' '''''' ' '
               uussssss----------------sssss----- - -----------------------sssss-------- - --------------ssssssss------ - -sssss------------- - --------ssssssuuuuuuuu u uuuuuuu u uuuuuu u u
                                                  ▽                                      ▽                              ▽                     ▽                 ▽      ▽         ▽        ▽
                                                  1                                      2                              3                     4                 5      6         7        8

                                         11111111 1 111111111111111111111111111111111111 1 1111111111111111122222222222 2 2222222222222222222 2 2222222222222333333333 3 3333333 3 333333 3 3
               5566777778888888888889999900001111 1 111111111222222222222222222344444444 5 5555555666666677700001111222 2 2223344445555555555 6 6666677789999000000000 1 1111222 2 222333 3 3
               7808455691122233368990124813671223 4 444566689001222223445556677511345669 1 2356678112446924588881558001 4 4581206880113455669 1 2257822362458011133667 0 1799224 4 555012 6 8
               6027703521223505759678792114849072 1 345625969085125788580683858035294191 1 6282307055035037501285458349 6 7299397497476801277 6 7949102408373056769455 9 7229571 3 147558 5 3

                                                                                                    11111111111111111111111 1 1111111111111111111 1 1111111112222222222222 2 2222222 2 222222 2 2
                  1111222222222233333333444555555 5 555556666666666666666666777788888899 9 9999900000001111133333334444 4 4455566777777777778 8 8889999990000111111111 2 2233333 3 333444 4 4
               1132888024455667702334567146005566 7 778990023345556667888999011845688902 4 6699902456770258900014781224 7 7714439013346777892 4 5570044573569122245778 2 2801335 5 666123 7 9
               1572258076778050204123247669394527 6 890170414530670233035138303580749646 6 1737852500580582056730903894 1 2744842942921356722 1 2494657919484167870566 0 8330682 4 258669 6 4

118958.Gmo.Ice .c...........................g.... . t.............g......t....c...g..... . ......a...c................a a a....c...t......... . .a..c..............ga. . ....... . t..cc. - .
152973.Gmo.Nor .c...........................g.... . t.g...........g......t....c...g..... . ......a...c................a a a....c...t......... . .a..c..............ga. . ....... . t...c. - .
200033.Gmo.Can .c...........................g.... . t.............g......t....c...g...c. . ......a...c................a a a....c...t......... . .a..c..............ga. . ....... . t...c. - .
126277.Gmo.Ice .c...........................g.... . t.............g......t...cc...g..... . ......a...c................a a a....c...t......... . .a..c..............ga. . ....... . t...c. - .
100957.Gmo.Gre .c...........................g.... . ..............g......t....c...g..... . ......a...c................a a a....c...t......... . .a..c..............ga. . ....... . t...c. - .
118708.Gmo.Ice .c...........................g.... . t.............g......t....c...g..... . ......a...c....g.....t.....a a a....ct..t......... . .a..c..............ga. . ....... . t...c. - .
117528.Gmo.Ice .c...........................g.... . t...........a.g...........c...g..... . ......a...c................a a a....c...t......... . .a..c..............ga. . ....... . t...c. - .
152978.Gmo.Nor .c......g....g..a............g.... . t...........a.g...........c...g..... . ......a...c................a a a....c...t......... . .a..c......g.......ga. . ....... . t...c. - .
118981.Gmo.Ice .c...........................g.... . t...........a.g...........c...g..... . ......a...c................a a a....c...t......... . .a..c..............ga. . ....... . t...c. - .
200203.Gmo.Can .c...........................g.... . t...........a.g...........c...g..... . ......a...c................a a a....c...t......... . .a..c..............ga. . ....... . t...c. - .
119411.Gmo.Ice .c...........................g.... . t...........a.g...........c...g..... . ......a...c................a a a....c...t......... . .a..c..............ga. . ....... . t...c. - .
140213.Gmo.Bar .c...........................g.... . t...........a.g...........c...g..... . ......a...c................a a a....c...t......... . .a..c..............ga. . ....... . t...c. - .
118335.Gmo.Ice .c.................a.........g.... . t...........a.g...........c...g..... . ......a...c................a a a....c...t......... . .a..c..............ga. . ....... . t...c. - .
140271.Gmo.Bar .c...........................g.... . t...........a.g...........c...g..... . ......a...c................a a a....c...t......... . .a..c..............ga. . ....... . t...c. . .
117937.Gmo.Ice .c.........................c.g.... . t...........a.g...........c...g..... . ......a...c................a a a....c...t......... . .a..c..............ga. . ....... . t...c. - .
152928.Gmo.Nor .c...........................g.... . t...........a.g...........c...g..... . ......a...c................a a a....c...t......... . .a..c..............ga. . ....... . t...c. - .
125372.Gmo.Ice .c...........................g.... . t...........a.g...........c...g..... . ......a...c................a a a....c...t......... . .a..c..............ga. . ....... . t...c. - .
```

continued on next page

```
                55eeeeeeiiiiiiiiiiiiiiiiieeeeeiiii i iiiiiiiiiiiiiiiiiiiiiiieeeeeiiiiiiii i iiiiiiiiiiiiiieeeeeeeeiiiiii i ieeeeeiiiiiiiiiiiii i iiiiiiiiieeeeee3333333 3 3333333 3 333333 3 3
                ''111111111111111111111112222222222 2 22222222222222222222222333333333333 3 33333333333333444555555555555 5 5666666666666666666 6 666666667777777''''''' ' ''''''' ' '''''' ' '
                uussssss-----------------sssss---- - -----------------------sssss-------- - --------------ssssssss------ - -sssss------------- - ---------sssssuuuuuuuu u uuuuuuu u uuuuuu u u
                                                  ▽                                    ▽                            ▽                   ▽               ▽       ▽       ▽      ▽
                                                  1                                    2                            3                   4               5       6       7      8

                                          11111111 1 111111111111111111111111111111111111 1 1111111111111111122222222222 2 2222222222222222222 2 2222222222222233333333 3 3333333 3 333333 3 3
                5566777778888888888889999900001111 1 111111112222222222222222222344444444 5 5555555666666677700001111222 2 2223344445555555555 6 6666677789999000000000 1 1111222 2 222333 3 3
                7808455691122233368990124813671223 4 444566689001222223445556677511345669 1 2356678112446924588881558001 4 4581206880113455669 1 2257822362458011133667 0 1799224 4 555012 6 8
                6027703521223505759678792114849072 1 345625969085125788580683858035294191 1 6282307055035037501285458349 6 7299397497476801277 6 7949102408373056769455 9 7229571 3 147558 5 3

                                                                                            11111111111111111111111 1 1111111111111111111 1 1111111122222222222222 2 2222222 2 222222 2 2
                   1111222222222223333333444555555 5 555556666666666666666677778888888999 9 9999000000011113333333334444 4 4455566777777777778 8 8889999990000111111111 2 2233333 3 333444 4 4
                1132888024455667702334567146005566 7 778990023345556667888999011845688902 4 6699902456770258900014781224 7 7714439013346777892 4 5570044573569122245778 2 2801335 5 666123 7 9
                1572258076778050204123247669394527 6 890170414530670233035138303580749646 6 1737852500580582056730903894 1 2744842942921356722 1 2494657919484167870566 0 8330682 4 258669 6 4

125430.Gmo.Ice  .c...........................g.....t...........a.g...........c...g...........a...c................aaa....c...t..........a..c..............ga.........t...c.-.
125859.Gmo.Ice  .c...........................g.....t...........a.g.........g.c...g...........a...c................aaa....c...t..........a..c..............ga.........t...c.-.
124471.Gmo.Ice  .c...........................g.....t...........a.g...........c...g...........a...c................aaa....c...t..........a..c..............ga.........t...c.-.
124406.Gmo.Ice  .c...........................g.....t...........a.g...........c...g...........a...c................aaa....c...t..........a..c..............ga.........t...c.-.
119204.Gmo.Ice  .c...........................g.....t...........a.g...........c...g...........a...c................aaa....c...t..........a..c..............ga.........t...c.-.
125848.Gmo.Ice  .c...........................g.....t...........a.g...........c...g...........a...c................aaa....c...t..........a..c..............ga.........t...c.-.
126003.Gmo.Ice  .c...........................g.....t...........a.g...........c...g...........a...c................aaa....c...t..........a..c..............ga.........t...c.-.
118970.Gmo.Ice  .c...........................g.....t...........a.g...........c...g...........a...c................aaa....c...t..........a..c..............ga.........t...c.-.
152921.Gmo.Nor  .c...........................g.....t...........a.g...........c...g...........a...c................aaa..t.c...t..........a..c..............ga.........t...c.-.
117868.Gmo.Ice  .c...........................g.....t...........a.g...........c...g...........a...c................aaa..t.c...t..........a..c..............ga.........t...c.-.
117801.Gmo.Ice  .c...........................g.....t...........a.g...........c...g...........a...c................aaa....c...t..........a..c..............ga.........t...c.-.
117757.Gmo.Ice  .c...........................g.....t...........a.g...........c...g...........a...c................aaa....c...t..........a..c..............ga.........t...c.-.
105746.Gmo.Gre  .c...........................g.....t...........a.g...........c...g...........a...c................aaa....c...t..........a..c..............ga.........t...c.-.
122879.Gmo.Ice  .c...........................g.....t...........a.g...........c...g...........a...c................aaa....c...t..........a..c..............ga.........t..cc.-.
122847.Gmo.Ice  .c...........................g.....t...........a.g...........c...g...........a...c................aaa....c...t..........a..c..............ga.........t..cc.-.
200397.Gmo.Can  .c...........................g.....t...........a.g...........c...g...........a...c............c...aaa.t..c...t..........a..c..............ga.....t...t...c.-.
125478.Gmo.Ice  .c...........................g.....t...........a.g...........c...g...........a...c................aaa....c...t.t........a..c..............ga.........t...c.-.
```

continued on next page

```
                 55eeeeeeeiiiiiiiiiiiiiiiiiieeeeeiiii i iiiiiiiiiiiiiiiiiiiiiiiieeeeeiiiiiiii i iiiiiiiiiiiiiiieeeeeeeeiiiiii i ieeeeeiiiiiiiiiiiiii i iiiiiiiiieeeeee33333333 3 3333333 3 333333 3 3
                 ''1111111111111111111111112222222222 2 2222222222222222222222222333333333333 3 33333333333333344455555555555 5 566666666666666666 6 66666666777777'''''''' ' ''''''' ' '''''' ' '
                 uusssssss----------------sssss---- - ------------------------sssss-------- - ----------------sssssss------ - -sssss------------------ - --------sssssuuuuuuuu u uuuuuuu u uuuuuu u u
                                                    ▽                                     ▽                                ▽                      ▽                   ▽       ▽         ▽        ▽
                                                    1                                     2                                3                      4                   5       6         7        8

                                          11111111 1 11111111111111111111111111111111111 1 1111111111111111112222222222 2 222222222222222222 2 2222222222222223333333 3 3333333 3 333333 3 3
                 556677777888888888888899999000011111 1 1111111111222222222222222222344444444 5 555555566666667770000111112222 2 222334444555555555 6 6666677789999000000000 1 1111222 2 222333 3 3
                 7808455691122233368990124813671223 4 444566689001222223445556677511345669 1 2356678112446924588881558001 4 4581206880113455669 1 2257822362458011133667 0 1799224 4 555012 6 8
                 6027703521223505759678792114849072 1 345625969085125788580683858035294191 1 6282307055035037501285458349 6 7299397497476801277 6 7949102408373056769455 9 7229571 3 147558 5 3

                                                                                                 1111111111111111111111 1 111111111111111111 1 111111111222222222222 2 2222222 2 222222 2 2
                    11112222222222233333333444555555 5 555555666666666666666677778888899 9 9999900000001111133333334444 4 445556677777777778 8 8889999990000111111111 2 2233333 3 333444 4 4
                 1132888024455667702334567146005566 7 778990023345556667888999011845688902 4 6699902456770258900014781224 7 7714439013346777892 4 5570044573569122245778 2 2801335 5 666123 7 9
                 1572258076778050204123247669394527 6 890170414530670233035138303580749646 6 1737852500580582056730903894 1 2744842942921356722 1 2494657919484167870566 0 8330682 4 258669 6 4
118324.Gmo.Ice.c.........................g.....t..........a.g..........c...g...........a...c..............aaa....c...t.t.........a..c.............ga.........t...c.-.
117879.Gmo.Ice.c.........................g.....t............g..........c...g...........a...c...............aa....c...t...........a..c.............ga.........t...c.-.
117896.Gmo.Ice.c.........................g.....t............g....c......ct..g...........a...c...............aa....c...t...........a..c.............ga.........t...c.-.
107799.Gmo.Gre.c.........................g.....t............g....c......c...g...........a...c...............aa....c...t...........a..c.........ggg..ga.........t...c.-.
117807.Gmo.Ice.c.........................g.....t............g....c......c...g...........a...c...............aa....c...t...........a..c.............ga.........t...c.-.
152047.Gma.Pac.c..........g....g...c......ggg........c...g....c.cg....t..c...g.g.......gtt....c.c...............gact.t....t..........gat.c...a...a.....g...g...t....tt....-a
100204.Gog.Gre.c..........g....gc..c......ggg........c...g......cg....t..c.c.g.g....g..gt.....c.c.g.........a...gact......t..........gat.c...a..........g.......t....tt....-a
152056.Gma.Pac.c..........g....gc..cg.....ggg........c...g....c.c.....t..c.c.g.g....g..gt.....c.c...............gact......t..........gat.c...a....t.....g.......t....tt....-a
152058.Gma.Pac.c..........g....g...c......ggg............g......cg....t..c.c.g.g....g..gt.....c.c...............gact......t..........gat.c...a....t.....g.......t....tt....-a
152064.Gma.Pac.c..........g....g...c......ggg............g......cg....t..c.c.g.g....g..gt.....c.c...............gact......t..........gat.c...a....t.....g.......t....tt....-a
152063.Gma.Pac.c..........g....g...c......ggg............g......cg....tg.c.c.g.g....g..gt.....c.c...............gact......t..........gat.c...a....t.....g.......t....tt....-a
152066.Gma.Pac.c..........g....g...c....c.ggg............g......cg....t..c.c.g.g....g..gt.....c.c...............gact......t..........gat.c...a....t.....g.......t....tt....-a
152035.Gch.Pac.c.t..c....t..at.g...c..a....g.g..-..........g.........t..t..c..gg.....c...gt..g..c.......................t..........ga.gc..a.g..........g....c.tt...tt....-a
104725.Bsa.Gre.c........c......g...c.......g.gga..g.a.c.c.ggt.c..c.....tt..c...ga..g.-...gt....gc..c..cggtt..a..a.........t.t......c..-ga..c.t.........--..g.g-....t.cctt....-a
104474.Bsa.Gre.c........c......g...c.......g.gga..g.a.c.c.ggt.c..c.....tt..c...ga..g.-...gt....gc..c.gcggtt.....a.........t.t......c..-ga..c.t.........--..g.g-....t.cttt....-a
```

continued on next page

55eeeeeeeiiiiiiiiiiiiiiiieeeeeiiii i iiiiiiiiiiiiiiiiiiiiiiiieeeeeiiiiiiii i iiiiiiiiiiiiiieeeeeeeeiiiiii i ieeeeeiiiiiiiiiiiii i iiiiiiiieeeeee33333333 3 3333333 3 333333 3 3
′′11111111111111111111111222222222 2 22222222222222222222222223333333333333 3 333333333333334445555555555 5 56666666666666666666 6 66666666777777′′′′′′′′ ′ ′′′′′′′′ ′ ′′′′′′′′ ′ ′
uussssss---------------sssss---- - ------------------------sssss-------- - --------------sssssss------- - -sssss------------- - --------sssssuuuuuuuu u uuuuuuu u uuuuuu u u
▽ ▽ ▽ ▽ ▽ ▽ ▽ ▽
1 2 3 4 5 6 7 8
11111111 1 111111111111111111111111111111111111 1 11111111111111111122222222222 2 2222222222222222222 2 22222222222222333333333 3 3333333 3 333333 3 3
5566777778888888888889999900001111 1 1111111112222222222222222222223444444444 5 5555555666666677700001111222 2 2223344445555555555 6 66666777899990000000000 1 1111222 2 222333 3 3
7808455691122233368990124813671223 4 444566689001222223445556677511345669 1 2356678112446924588881558001 4 4581206880113455669 1 22578223624580111336670 0 1799224 4 555012 6 8
6027703521223505759678792114849072 1 345625969085125788580683858035294191 1 6282307055035037501285458349 6 7299397497476801277 6 79491024083730567694559 9 7229571 3 147558 5 3
1111111111111111111111 1 111111111111111111 1 11111111122222222222222 2 2222222 2 222222 2 2
1111222222222223333333344455555 5 5555556666666666666666666777788888899 9 999990000000111113333333344444 4 4455566777777777778 8 8889999999000011111111 2 2233333 3 333444 4 4
1132888024455667702334567146005566 7 778990023345556667888999011845688902 4 6699902456770258900014781224 7 7714439013346777892 4 55700445735691222457778 2 2801335 5 666123 7 9
1572258076778050204123247669394527 6 890170414530670233035138303580749646 6 1737852500580582056730903894 1 2744842942921356722 1 24946579194841678705660 0 8330682 4 258669 6 4

[a]The top panel of the boxhead indicates 5′ UTR, exon, intron, and 3′ UTR location of segregating sites, numbers represent exon and intron numbers, s, n, -, u, and ∇ represent synonymous, nonsynonymous, intronic, UTR, and indel sites respectively. The middle panel of the boxhead are site numbers of the *Ckma* gene (3604 bp) and the bottom panel are site numbers of the concatenated 2500 bp sequenced fragment (Supplementary Figure S2). Horizontal lines are drawn between *A* and *B* alleles and between Atlantic cod and closely related species Gma *Gadus macrocephalus*, Gch *Gadus chalcogrammus*, Gog *Gadus ogac*, and Bsa *Boreogadus saida*. Locality codes are as indicated in methods. ∇_1 is a four bp indel at sites 573–576. ∇_2 is an 11 bp indel at sites 941–951. ∇_3 is a two bp indel at sites 1470–1471. ∇_4 is an 14 bp indel at sites 1828–1841. ∇_5 is an three bp indel at sites 2127–2129. ∇_6 is a one to eight bp indel at sites 2217–2224. ∇_7 is a one bp indel at site 2354. ∇_8 is a one bp indel at site 2476. The Moen et al. Gm366-0514 locus with an $F_{ST} = 0.83$ is at site 1634, the Gm366-1022 locus with an $F_{ST} = 0.82$ is at site 2426, and the Gm366-1073 with an $F_{ST} = 0.82$ is at site 2476. *A* alleles are in red and *B* alleles in blue. Closely related species are colored: Gma is green, Gog is darkgreen, Gch is darkred, and Bsa is darkblue.

Finished
